# Gynecomastia on Thoracic Computed Tomography

**DOI:** 10.7759/cureus.51509

**Published:** 2024-01-02

**Authors:** Muhammed Akif Deniz, Rukan Matsar Öz

**Affiliations:** 1 Radiology, Dicle University School of Medicine, Diyarbakır, TUR; 2 Radiology, Diyarbakır Pediatric Disease Hospital, Diyarbakır, TUR

**Keywords:** cancer, male breast imaging, thorax, computed tomography, gynecomastia

## Abstract

Background and objective

Gynecomastia is a benign proliferation of ductal epithelium in the retroareolar region in male patients. The aim of this study was to investigate the frequency of gynecomastia in male patients who underwent thoracic computed tomography (CT) imaging at our clinic, assess possible causes, highlight the imaging characteristics of gynecomastia, and compare our findings with the literature.

Materials and methods

Male patients over 18 years of age who underwent thoracic CT imaging in our clinic were included in the study. Patients were initially assessed based on age and the presence of gynecomastia. The patients with gynecomastia were evaluated in terms of age, gynecomastia localization (right, left, and bilateral), gynecomastia type (nodular, dentritic, and diffuse), and possible etiology.

Results

The study included 1500 patients with a mean age of 45.6±21.7 years, and 470 (31.3%) patients had gynecomastia. Gynecomastia was on the right side in 11.3%, on the left side in 11.1%, and bilateral in 77.7% of the patients. Gynecomastia was nodular in 52.1%, dendritic in 35.3%, and diffuse in 17.2% of the patients. The causative factor could not be identified in 44.3% of the patients with gynecomastia. Among cases where the etiology was identified (56.7%), the most common factors were cancer (23.4%), chronic kidney disease (CKD) (13.2%), and chronic hepatitis B (10.7%).

Conclusion

When evaluating thoracic CT, the breast area, in addition to the lungs, chest wall, and bone structures, should also be evaluated carefully. With the increased use of thoracic CT scans, incidentally detected gynecomastia in patients is also on the rise. Knowing the presence of gynecomastia is very important for the clinician to determine the etiology and treat the underlying disease. Therefore, detecting and reporting gynecomastia on thoracic CT can prevent unnecessary advanced breast imaging methods and play a very important role in treating the underlying etiology.

## Introduction

Gynecomastia is the benign proliferation of ductal epithelium in the retroareolar region in male patients and is the most common breast condition in males. While there are multiple etiological factors, it is primarily believed to result from hormonal imbalance involving various mechanisms. Physiological gynecomastia is generally observed in three periods. In the neonatal period, it can be seen in 60%-90% of the cases due to the influence of hormones transmitted from the mother, typically resolving spontaneously within 2-4 weeks after birth. The second period is during adolescence, thought to arise from an imbalance between androgens and estrogens. The third period is in older age, where a decrease in androgens is considered to play a role [1-4]. The etiology of pathological gynecomastia can be attributed to certain medications (such as aldosterone antagonists, antiulcer drugs, certain chemotherapeutics, some cardiovascular drugs, certain antipsychotics, and hormone medications) and certain medical conditions (liver cirrhosis, hormone-secreting testicular tumors, chronic kidney diseases {CKD}, lung cancer, paraneoplastic syndrome, primary-secondary hypogonadism, hyperthyroidism, etc.) [2-5].

The primary goal in evaluating breast enlargement in males is to differentiate gynecomastia from other conditions such as pseudogynecomastia, which is associated with obesity and manifests as an increase in fatty tissue within the breast, as well as gynecomastia from breast cancer or isolated enlargement of the nipple. Diagnosis commonly involves physical examination, ultrasound (US), and mammography. While computed tomography (CT) is not typically used for diagnosing gynecomastia, the presence of gynecomastia can be easily detected in patients undergoing thoracic CT because the breast tissue is completely in the field of view [6-8].

The aim of this study was to investigate the frequency of gynecomastia in male patients undergoing thoracic CT imaging in our clinic, assess possible causes, highlight the imaging characteristics of gynecomastia, and compare our findings with the literature.

## Materials and methods

This study was designed as a retrospective archival study. Male patients over 18 years of age who underwent thoracic CT imaging between 01/01/2022 and 16/02/2023 in our clinic were included in the study. Patients were initially assessed based on age and the presence of gynecomastia. The patients with gynecomastia were evaluated in terms of age, gynecomastia localization (right, left, and bilateral), gynecomastia type (nodular, dendritic, and diffuse), and possible etiology. The ethical approval for the study was obtained from the Ethics Committee for Non-Interventional Clinical Research, Faculty of Medicine, Dicle University (approval number: 244; date: 09/06/2022).

In the present study, the definition and categorization of gynecomastia were based on those described in the literature. Accordingly, patients with glandular tissue measuring 2 cm or more in the breast tissue are considered to have gynecomastia, which has three types. Nodular gynecomastia is the most common and is histologically known as the florid phase. The glandular tissue takes on a fan-shaped form, spreads from the nipple, and gradually blends into the surrounding fat tissue. Sometimes, this density appears opaque and resembles a sphere. Dendritic gynecomastia is less common and corresponds to a fibrous structure. It is seen as projections (dendrites) that irradiate and penetrate the periphery, and there is a presence of tissue that can extend to the upper external quadrant. Diffuse gynecomastia is the less common form characterized by a significantly high density of breast tissue, resembling the appearance of female breasts [6-10].

Computed tomography protocol

All examinations were conducted using a 64-detector CT scanner (Philips Brilliance 64 Channel, Philips Healthcare, Eindhoven, Netherlands). The imaging parameters were set as follows: 120 kVp, 300 mAs, 1 mm slice thickness, 0.5 pitch, and a 220 mm field of view. All images were sent to the picture archiving and communication system (PACS) to generate multiplanar images and evaluated. All images were evaluated by two radiologists with at least five years of experience in thoracic-breast imaging. In cases where there was a difference of opinion between the two radiologists, the images were re-evaluated together until a consensus was reached. The images were evaluated in the axial plane, and the measurement of glandular tissue was performed in the axial plane. For patients with multiple scans, one artifact-free scan with clearly distinguishable borders of breast tissue was included in the study.

Patients with the incomplete visualization of breast tissue, images affected by artifacts, patients who underwent breast surgery, individuals with a history of trauma at the level of the breast area, and patients with breast masses were excluded from the study. Additionally, high-resolution CT (HRCT) scans were also excluded due to the lower soft tissue resolution at the level of the breast region (an average of 500 patients were excluded).

Statistical analysis

Analyses were performed using the Statistical Package for Social Sciences (SPSS) version 22 software package (IBM SPSS Statistics, Armonk, NY). Descriptive data were presented as n (%) for categorical variables and as mean±standard deviation (mean±SD) for continuous variables. The comparison of categorical data was conducted using the chi-square test. The normal distribution of continuous variables was assessed using the Kolmogorov-Smirnov test. The Mann-Whitney U test was used to assess differences between two independent groups, while the Kruskal-Wallis test was used for comparisons involving more than two groups. The Spearman correlation test was used to examine the relationship between continuous variables. A p-value of less than 0.05 was considered statistically significant in the analyses.

## Results

**Figure 1:**
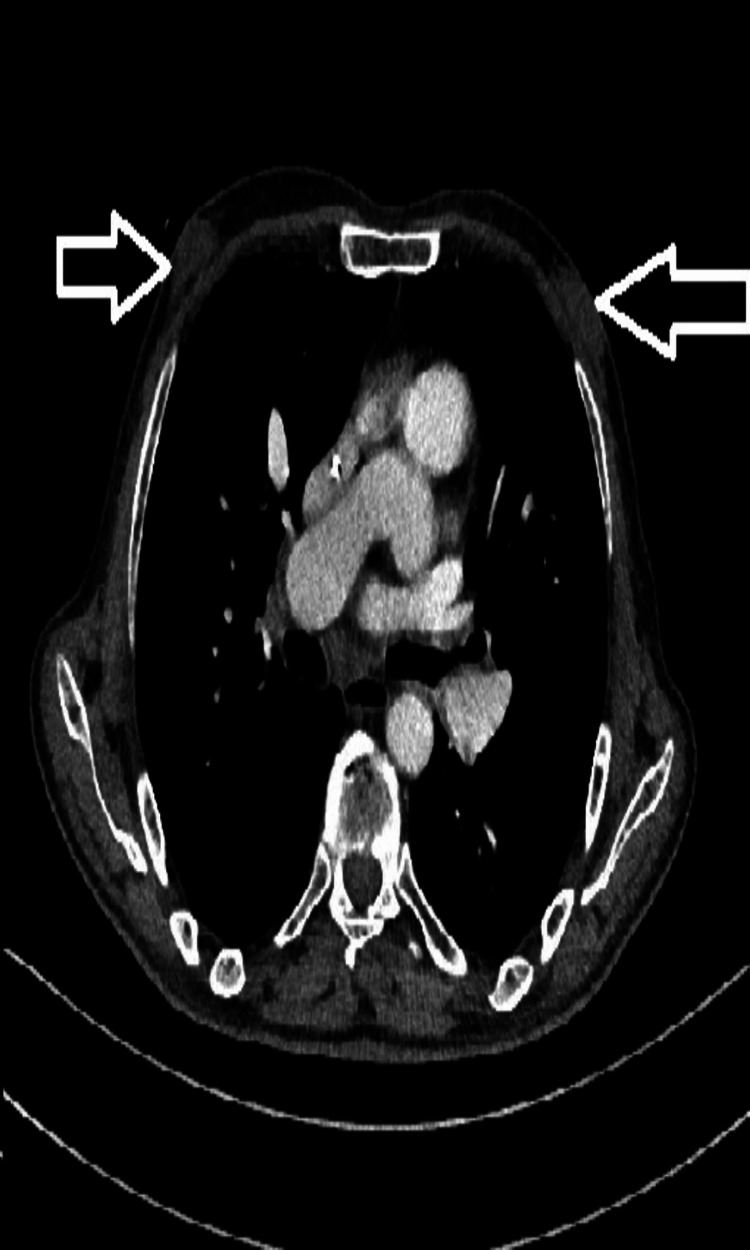
A 61-year-old patient with chronic kidney failure and dendritic-type gynecomastia in the retroareolar region of both breasts (marked with an arrow)

**Figure 2:**
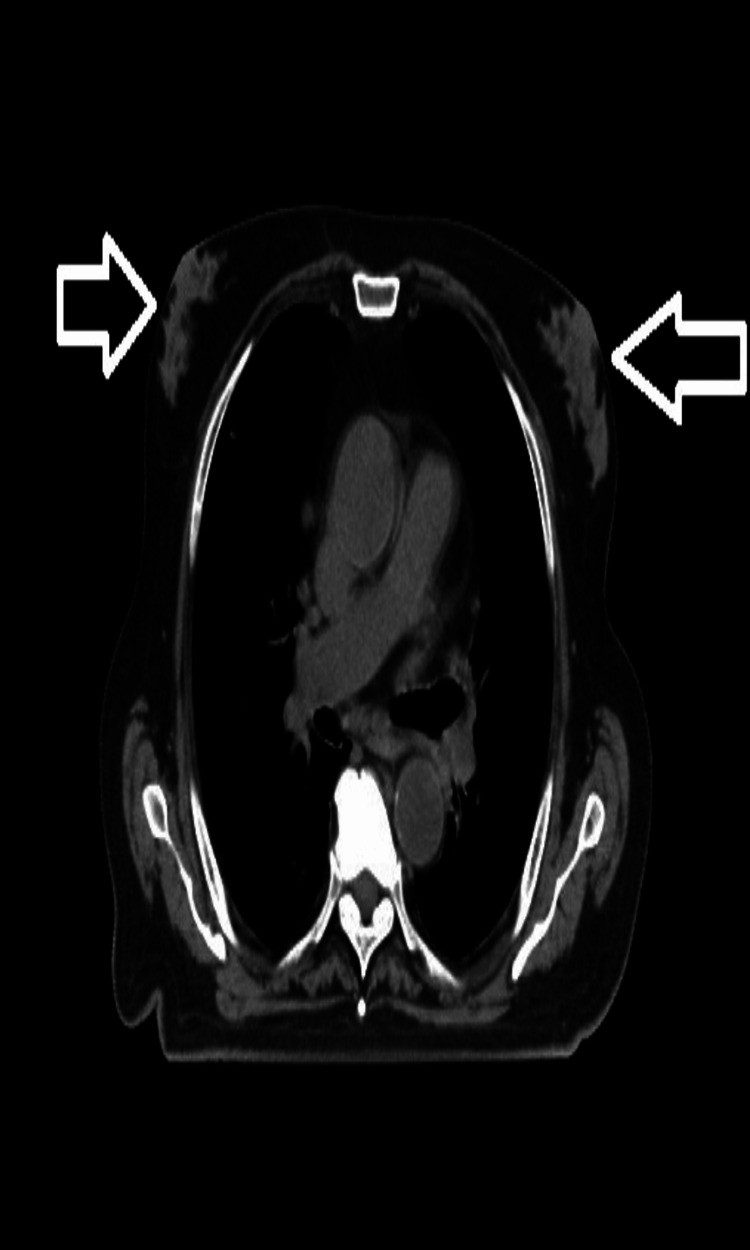
A 76-year-old patient with prostate cancer and diffuse-type gynecomastia in the retroareolar region of both breasts (marked with an arrow)

**Figure 3:**
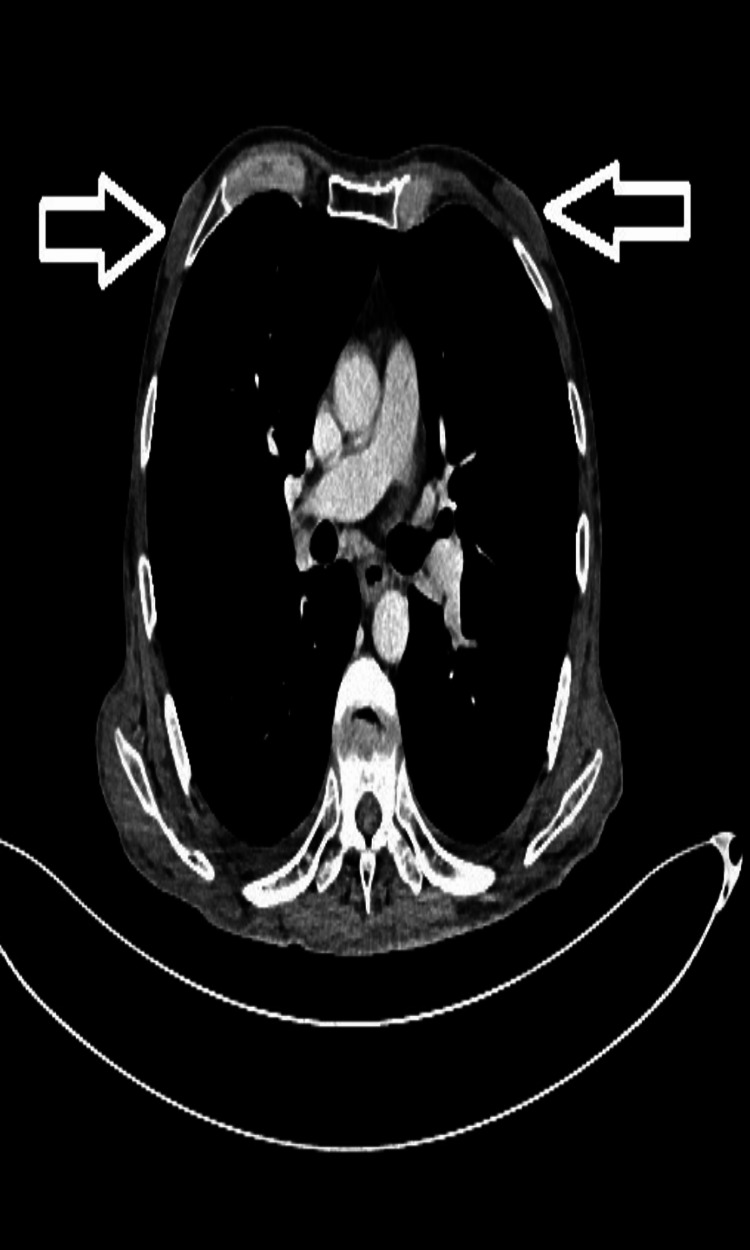
A 53-year-old patient with chronic hepatitis B and nodular-type gynecomastia in the retroareolar region of both breasts (marked with an arrow)

The study included 1500 patients with a mean age of 45.6±21.7 years (minimum=18 and maximum=86). Among all patients, 470 (31.3%) had gynecomastia. Gynecomastia was on the right side in 11.3%, on the left side in 11.1%, and bilateral in 77.7% of the cases. The size of gynecomastia was measured as 26.1±8.1 mm on the right side and 26.5±7.9 mm on the left side. Of those with gynecomastia, 52.1% had a nodular type, 35.3% had a dendritic type, and 17.2% had a diffuse type (Table 1) (Figures 1-3).

**Table 1:** Characteristics of males with gynecomastia *Includes patients with more than one type of gynecomastia SD: standard deviation

		Number	%
Laterality	Right	53	11.3
	Left	52	11.1
	Bilateral	365	77.7
Right gynecomastia size, mean±SD		26.1±8.1	
Left gynecomastia size, mean±SD		26.5±7.9	
Type of gynecomastia*	Nodular	245	52.1
	Dendritic	166	35.3
	Diffuse	81	17.2

Among those with gynecomastia on the right side, 51.9% had a nodular type, while 52.4% of patients with gynecomastia on the left side had a nodular type, and no significant difference was observed between the groups (p=0.887). Additionally, 34.4% of those with gynecomastia on the right side had a dendritic type, and 34.9% of those with gynecomastia on the left side had a dendritic type, with no significant difference between them (p=0.902). Furthermore, a diffuse type was seen in 18.9% of patients with gynecomastia on the right side and 17.5% of those with gynecomastia on the left side, with no significant difference between the groups (p=0.613). There was no significant difference in gynecomastia size between the right and left sides (p=0.151) (Table 2).

**Table 2:** Comparison of gynecomastia type and size according to laterality *Chi-square analysis; **Mann-Whitney U test SD: standard deviation

		Right		Left		p*
		Number	%	Number	%	
Nodular	Present	217	51.9	218	52.4	0.887
	Absent	201	48.1	198	47.6	
Dendritic	Present	144	34.4	145	34.9	0.902
	Absent	274	65.6	271	65.1	
Diffuse	Present	79	18.9	73	17.5	0.613
	Absent	339	81.1	343	82.5	
Gynecomastia size, mean±SD		26.1±8.1		26.5±7.9		0.151^**^

The mean age of males with gynecomastia was significantly higher than the mean age of males without gynecomastia (p=0.007). The mean age of those with nodular-type gynecomastia was significantly lower than the mean age of those without (p=0.025). The mean age of those with diffuse-type gynecomastia was significantly higher than the mean age of those without (p<0.001). There was no significant age difference with respect to laterality (p=0.555) and the presence of dendritic-type gynecomastia (p=0.558) (Table 3).

**Table 3:** Comparison of age according to the presence of gynecomastia *Mann-Whitney U test; **Kruskal Wallis test SD: standard deviation

		Age	p*
		Mean±SD	
Gynecomastia	Present	52.3±20.7	0.007
	Absent	48.4±22.0	
Laterality	Right	53.1±20.2	0.555^**^
	Left	55.0±20.2	
	Bilateral	51.8±20.8	
Nodular	Present	50.0±22.7	0.025
	Absent	54.8±18.0	
Dendritic	Present	53.3±17.6	0.558
	Absent	51.7±22.2	
Diffuse	Present	59.8±18.0	<0.001
	Absent	50.7±20.9	

There was a significant positive correlation between age and right- and left-side gynecomastia size (Table 4).

**Table 4:** Correlation of gynecomastia size with age

	Age	
	r	p
Right-side gynecomastia size	110	024
Left-side gynecomastia size	156	001

The causative factor could not be identified in 44.3% of gynecomastia patients. Among cases where the etiology was identified (56.7%), the most common factors were cancer (23.4%) (the first five most common cancers are lung cancer, colon cancer, stomach cancer, larynx cancer, and rectum cancer) chronic kidney disease (CKD) (13.2%), chronic hepatitis B virus (HBV) (10.7%), and medication (8.4%) (the most common causes are aldosterone antagonists, antiandrogenic drugs, antiulcer drugs, some chemotherapeutics, and some cardiovascular drugs) (Table 5).

**Table 5:** Possible causes of gynecomastia CKD, chronic kidney disease; HBV, hepatitis B virus

	Number	%
Not detected	208	44.3
Cancer	110	23.4
CKD	62	13.2
Chronic HBV	50	10.7
Medication	40	8.4

## Discussion

Gynecomastia is a benign proliferation of glandular tissue and affects approximately one-third of males [11-15]. It is mostly incidentally detected on thoracic CT scans. In our study investigating the incidence of gynecomastia on thoracic CT scans, we observed a gynecomastia rate of 31.3%. The reported prevalence of gynecomastia in the literature varies between 32% and 65% [2,11,12]. Kim et al. [4] identified gynecomastia in 12.7% of the cases in their study. Gossner [13] reported a gynecomastia rate of 25.6% in his study. In a study conducted by Aslan et al. [14] involving 1877 patients, the incidence of gynecomastia was found to be 32.3%. The frequency of gynecomastia observed in our study is comparable to that reported in the study by Aslan et al. [14].

Although gynecomastia can sometimes occur unilaterally, it is generally bilateral [1,12,14]. The prevalence of unilateral gynecomastia in patients was found to be 14% in a previous mammography-based study [7]. Kim et al. [4] observed unilateral gynecomastia in 36.8% of the cases in their study. Gossner [13] reported unilateral gynecomastia in 4.7% of the cases he examined. Aslan et al. [14] found unilateral gynecomastia with a rate of approximately 25% in their study. In the same study, right and left gynecomastia were observed at similar rates in patients with unilateral gynecomastia. In our study, similar to the literature, unilateral gynecomastia was observed in 23% of the patients. Furthermore, the occurrence of gynecomastia on the right and left sides was observed at similar rates (11.3% and 11.1%, respectively).

Gynecomastia has been classified into three types based on the prevalence and size of glandular tissue. The nodular pattern is considered the acute phase of gynecomastia, while the dendritic phase is described as a more widespread form. The diffuse type exhibits more prominent glandular tissue and resembles a female-type breast structure. In the literature, the nodular type has been described as the most common type, while the diffuse type has been reported as the least common type [6-8]. In a study by Aslan et al. [14], the dendritic type was identified as the most frequent, while the diffuse type was identified as the least frequent type. In a study by Kim et al. [4], the most common type observed was the nodular type, and the least common was the diffuse type. Similarly, in our study, consistent with Kim et al.'s study [4], the most frequent type of gynecomastia observed was the nodular type, and the least frequent was the diffuse type. In addition, there was no statistically significant difference between the type of gynecomastia and right and left localization in our study.

Studies describing the relationship between the presence of gynecomastia, gynecomastia type, and age are quite limited in the literature. In a study by Aslan et al., no significant difference was found in terms of age between patients with and without gynecomastia [14]. In the present study, the mean age of males with gynecomastia was significantly higher than the mean age of males without gynecomastia. Additionally, the mean age of those with nodular-type gynecomastia was found to be significantly lower than the mean age of those without. Conversely, the mean age of males with diffuse-type gynecomastia was significantly higher than the mean age of those without. There was no significant difference in age with respect to lateralization and the presence of dendritic-type gynecomastia.

According to the literature, the cutoff value for glandular tissue thickness in gynecomastia is reported as 2 cm or above [6-8]. In the present study, patients with glandular tissue thickness equal to or greater than 2 cm were considered positive for gynecomastia. In the study by Kim et al. [4], the mean glandular tissue thickness was found to be 2.5 cm (ranging from 2.2 to 3.1 cm). Klang et al. [11] reported an average glandular tissue thickness of 2.2 cm in their study. In the present study, the size of gynecomastia was 26.1 mm on the right side and 26.5 mm on the left side, with an average size of 26.3 mm. Additionally, a positive significant correlation was observed between patient age and the size of gynecomastia on the right and left sides.

Gynecomastia is mostly asymptomatic. Various medications and chronic illnesses play a role in its etiology. Primary or secondary hypogonadism, testicular tumors, hyperthyroidism, liver cirrhosis, and chronic kidney disease are among the most common causes. The increase in gynecomastia prevalence in recent years has also been associated with the use of anabolic steroids by athletes. Studies in the literature have documented cases where the underlying cause could not be fully identified [3-8]. In his study, Gossner [13] was able to identify the etiology in 95% of the cases. However, the number of patients in this study was relatively low. In the present study, the etiology was identified in 55.7% of the patients diagnosed with gynecomastia, with cancer, chronic kidney disease (CKD), and chronic hepatitis B being the most frequent causes. We think that steroids used by athletes may be one of the causes of gynecomastia in patients whose etiology could not be determined in our study.

Our study has some limitations. The most important limitation of the study is its retrospective nature. Additionally, the relatively small number of patients and the inability to correlate our results with ultrasound and mammography findings are among the limitations of our study.

## Conclusions

As a result, in our study where we investigated the frequency of gynecomastia in thoracic CT, nodular and bilateral gynecomastia were detected most frequently. When evaluating thoracic CT, the breast area, in addition to the lungs, chest wall, and bone structures, should also be evaluated carefully. With the increased use of thoracic CT scans, incidentally detected gynecomastia in patients is also on the rise. Knowing the presence of gynecomastia is very important for the clinician to determine the etiology and treat the underlying disease. Therefore, detecting and reporting gynecomastia on thoracic CT can prevent unnecessary advanced breast imaging methods and play a very important role in treating the underlying etiology.
